# Research Progress on the Application of CGM in Patients with Diabetes and Hemodialysis

**DOI:** 10.7150/ijms.102727

**Published:** 2024-11-11

**Authors:** Ping Huang, Jing He, Liansheng Ren, Rong Yang, Dan Feng, Ling Li, Shuhuan Liu, Yunmin Wang, Yi Zeng, Wei Zhang, Dan Zhu

**Affiliations:** 1 363 Hospital, 108 Daosangshu Street, Wuhou District, Chengdu, 610000, People's Republic of China.; 2 Center for Endemic Disease Control, Chinese Center for Disease Control and Prevention, Harbin Medical University, Harbin, 150081, People's Republic of China; National Health Commission & Education Bureau of Heilongjiang Province, Key Laboratory of Etiology and Epidemiology, Harbin Medical University (23618504), Harbin, 150081, People's Republic of China; Center for Chronic Disease Prevention and Control, Harbin Medical University, Harbin, People's Republic of China.

**Keywords:** Continuous Glucose Monitoring, Diabetes Mellitus, Hemodialysis, Blood glucose fluctuation

## Abstract

Diabetes mellitus is the main cause of end-stage renal disease (ESKD), and most patients need hemodialysis (HD) treatment after they progress to uremia. Patients with diabetes and HD have obvious blood glucose fluctuation, hyperglycemia and hypoglycemia may both related to the higher mortality. Therefore, maintaining blood glucose stability is the main treatment strategy to improve the prognosis of patients. It is challenging to evaluate the blood glucose control of patients with diabetes and HD. The traditional blood glucose detection methods have certain limitations, they may be affected by many factors in HD patients. The application of continuous glucose monitoring (CGM) system is gradually recognized, CGM can monitor blood glucose real-time, timely, and predictive capabilities, there are fewer factors that are affected blood glucose in HD patients.

## Introduction

The International Diabetes Federation estimates that the number of people with diabetes will reach 784 million by 2045. Diabetes is a major cause of chronic kidney disease (CKD), and many patients end up with renal failure and require hemodialysis (HD) 1-3. Studies have shown that rapid blood glucose fluctuations in HD patients can lead to microvascular and macrovascular diseases 4, 5. Traditional blood glucose monitoring methods have many factors affecting their accuracy. CGM provides more timely and accurate data, which is important for diabetes and HD patients 6. This article will review the research progress related to the application of CGM in patients with diabetes and HD.

## Clinical challenges

### Characteristics of blood glucose fluctuation in HD patients

Studies have confirmed that diabetes patients receiving hemodialysis have significant blood glucose fluctuations and a high incidence of hypoglycemia. Factors include uremia reducing gluconeogenesis, renal dysfunction weakening insulin clearance, dialysis causing malnutrition, increased red blood cell glucose uptake, rapid plasma glucose removal by dialyzer, abnormal secretion of anti-regulatory hormones, improved insulin sensitivity after dialysis, and exogenous insulin application leading to mostly asymptomatic and dangerous hypoglycemia 6-10. Hypoglycemia during hemodialysis can lead to post-dialysis hyperglycemia and increased blood glucose drift by stimulating anti-regulatory hormones. In HD patients, renal dysfunction causes insulin accumulation and insulin resistance, forming a vicious cycle. The dialyzer membrane can adsorb plasma insulin and increase blood glucose 7, 11. In HD patients, hypoglycemia and hyperglycemia may happen during and after HD, making blood glucose changes hard to predict. Hypoglycemia raises the risk of cognitive dysfunction and can cause fatal arrhythmia. Severe hypoglycemia may lead to coma or death, and the risk of hypoglycemia events grows with intensive blood glucose treatment 11-13. Post-HD hyperglycemia causes oxidative stress, inflammation, cell death, and increased glucose toxicity, leading to clinical symptoms and potentially being life-threatening 7. Excessive glucose fluctuation may be more dangerous than persistent abnormal blood glucose. GV is related to microvascular and macrovascular complications, hypoglycemia, and a higher risk of death 14, 15.

### Limitations of traditional blood glucose monitoring

Diabetes patients who maintain HD need effective blood glucose control. Hyperglycemia, hypoglycemia and acute blood glucose fluctuation are all related to high mortality 45-47. Among them, SMBG, HbA1c and GA are commonly used to monitor the blood glucose level of ordinary patients with diabetes, and there may be some limitations in monitoring the blood glucose level of patients with diabetes and HD 13, 16.

SMBG is the most commonly used method, which is generally used to monitor blood glucose before meals, two hours after meals and before going to bed. It has certain limitations, including the discomfort caused by acupuncture blood collection, the accuracy of blood glucose measurement results may be affected by the precision of blood glucose meter, contaminated test paper and irregular operation 4. SMBG only provides monitoring the blood glucose level at that moment, but does not obtain enough blood glucose data to reflect the daily blood glucose control, daily GV and the occurrence of nocturnal and asymptomatic hypoglycemia. It has limitations in evaluating the daily glucose fluctuation in detail to guide treatment, and the blood glucose data monitored by SMBG may not be able to make appropriate treatment decisions 17. However, CGM can continuously monitor blood glucose levels throughout the day, providing a more comprehensive understanding of blood glucose fluctuations than intermittent SMBG. It can detect changes in blood glucose in real time and alert patients and healthcare providers to potential hypoglycemic or hyperglycemic events earlier than SMBG. CGM reduces the burden on patients of frequent finger pricks. It can provide more detailed data on glucose variability, helping healthcare providers better manage and adjust treatment plans. The convenience and continuous monitoring of CGM can improve patient compliance with treatment 27.

HbA1c is the gold standard for evaluating the long-term blood glucose control level of diabetes mellitus, which is used to estimate the average blood glucose level of patients for 2 to 3 months. The KDIGO guidelines provide glycosylated hemoglobin targets for chronic kidney disease stage 1-5, but these targets are not suitable for HD people 13. HbA1c is influenced by many factors during HD, resulting in unreliable results. The formation of HbA1c depends on the intensity and duration of non-enzymatic interaction between blood glucose and hemoglobin, so drugs that change erythropoiesis and longevity will affect the value of HbA1c 16. The measured HbA1c value of patients with diabetes and HD may be lower than the actual value due to many factors, such as shortened life span of red blood cells, blood transfusion, use of iron and erythropoietin, while the measured HbA1c value is higher when there is iron deficiency or VitB12. At the same time, uremia environment in HD patients will stimulate carbamylation of hemoglobin, which will also interfere with the determination of HbA1c by ion exchange method 18. The research by Galindo RJ et al. found that when using CGM to monitor the blood glucose level of type 2 diabetes patients on hemodialysis, the results showed that GMI has a strong correlation with TIR, while HbA1c underestimates the patient's MG and GMI 57. With respect to HbA1c, CGM can reflect recent changes in blood glucose, can visualize blood glucose fluctuations 57. Strikingly, shortened red blood cell lifespan, blood transfusion, use of iron and erythropoietin, iron deficiency or vitamin B12 deficiency, and uremic environment do not have an impact on the results of CGM 18.

Although the measurement result of GA will not be affected by the influencing factors of HbA1c, if hypoproteinemia occurs, the result of GA may also be unreliable, because the degree of glycosylation of albumin is determined by the reaction time between albumin and blood glucose 19, 20. As for GA, CGM is not affected by hypoproteinemia, can monitor blood glucose in a timelier fashion and provide more detailed information.

## Overview of CGM

American Diabetes Association (ADA) and Guidelines for Blood Glucose Monitoring in China recommend that most patients with type 1 diabetes and some patients with type 2 diabetes use CGM as an important supplementary means of blood glucose monitoring. CGM is especially suitable for patients with high blood glucose fluctuation and high risk of hypoglycemia, especially those with repeated nocturnal hypoglycemia and non-conscious hypoglycemia 21. CGM includes retrospective CGM, real-time CGM and scanning CGM, in which real-time CGM can monitor the blood glucose level of patients in real time and record up to 280 measurements every day (usually once every 1-5 minutes). CGM collects the blood glucose level for 24 hours and displays it in the form of ambulatory glucose profile (AGP), which can concisely and intuitively present the blood glucose level, blood glucose fluctuation, hypoglycemia or hyperglycemia of patients. Realizing the visualization of "blood glucose triangle" can not only provide real-time data about blood glucose level and trend, but also provide retrospective data about blood glucose control mode in specified time period and blood glucose index, which can accurately reflect blood glucose and control level 17. It is undeniable that the use of CGM has certain limitations: firstly, the cost of CGM is higher; secondly, CGM detection value delayed 15-20 minutes, compared with the fingertip blood glucose value; thirdly, the loss of sensor and mobile device signals during the use of CGM may affect the timely monitoring of blood glucose levels by patients and medical staff.

The KDIGO guidelines has clearly pointed out that for patients with diabetes and chronic kidney disease (CKD), especially those with large blood glucose fluctuations and difficult to effectively control blood glucose through traditional blood glucose monitoring methods, using continuous glucose monitoring (CGM) can avoid further damage to the kidneys caused by too high or too low blood glucose 3. The ADA guidelines further refine the blood glucose control targets for different patient groups according to the data provided by CGM and emphasize the close combination of CGM data and treatment plans 22. Chinese guidelines have also taken CGM as an important blood glucose monitoring method, which can provide accurate blood glucose data and adjust treatment plans, dietary structures and exercise methods 50. The level of blood glucose control is expressed using standardized CGM indicators such as time in range (TIR) (3.9-10.0 mmol/L), time above range (TAR) Level 1 (10.1-13.9 mmol/L) and level 2 (>13.9 mmol/L), Time below range (TBR) Levels 1 (3.0-3.8 mmol/L) and 2 (<3.0 mmol/L), glucose management Indicators (GMI), mean glucose (MG), and Glucose Variability (GV) 17, 23. Among them, TIR is one of the key indicators of CGM. For general patients with diabetes, the main objective of effective and safe blood glucose control is to increase TIR to more than 70%. However, for patients with diabetes with high risk factors including dialysis, it is recommended to lower the target of TIR to >50% in order to reduce the incidence of hypoglycemia. It decreased to less than 1% at TBR (<3.9mmol/L), less than 50% at TAR (>10.0mmol/L), and less than 10% at TAR (>13.9mmol/L) 13, 16, 23. GMI is an indication of the current state of a person's glucose management, 10-14 days of CGM blood glucose data can be used to estimate CGM indicators within 3 months, can produce a representative CGM-derived average glucose value, based on this average glucose, and using a standard formula can produce a value known as "estimated HbA1c" (HbA1c). In the absence of factors affecting the lifespan of red blood cells, the value of HbA1c measured in the laboratory was approximately the same 24. GV is also one of the important indicators for CGM to monitor blood glucose level, which is usually defined as the frequency and amplitude of blood glucose oscillation or the change of acute blood glucose. GV can better reflect the situation of blood glucose control than HbA1c 25.

## Applications of CGM in patients with diabetes HD

There have been a number of studies on the application of CGM in patients with diabetes HD, including exploring the blood glucose profile, incidence of hypoglycemia and blood glucose fluctuations in patients with diabetes HD, evaluating the accuracy of traditional blood glucose monitoring methods in patients with diabetes HD, and the efficacy and safety of anti-hyperglycemic drugs (Table 1).

### Exploring the blood glucose spectrum, incidence of hypoglycemia and blood glucose fluctuations

CGM has been widely used to explore the blood glucose spectrum of patients with diabetes and HD. Studies have found that patients with diabetes and HD have significant daytime GV, and their blood glucose fluctuates more significantly on dialysis days than on non-dialysis days. With the same energy intake, the average glucose level during HD is significantly lower than that on non-dialysis days, and the risk of hypoglycemia is increased 30, 41, 49. This may be because glucose metabolism in patients with diabetes and HD is affected by a variety of factors, including insulin secretion and degradation, insulin resistance, altered drug metabolism, increased inflammatory markers, protein catabolism, anti-regulatory hormone secretion, use of no or low glucose dialysate, glucose loss during dialysis, and decreased renal gluconeogenesis. All of these factors affect GV 12.

Studies have confirmed that the frequency of hypoglycemia in patients with diabetes and HD is higher, and the reduced level of kidney function may be closely related to the risk of hypoglycemia in patients 31. Most studies that when using CGM to monitor the blood glucose of diabetic patients on HD, compared with non-HD days, TIR of patients on HD days is reduced and TBR is increased. The blood glucose fluctuation on HD days is higher than that on non-HD days, especially from the start of hemodialysis to two hours after hemodialysis. GV of HD patients is also higher than that of non-HD patients 49, 56. Most of the patients reached the lowest glucose level (79mg/dl) in the first 3 hours after the beginning of dialysis, and the postdialysis hyperglycemia occurred 2.5 hours after the end of dialysis, and the interstitial glucose concentration could reach 187mg/dl 29. Compared with HD patients without diabetes, the blood glucose fluctuation in HD patients with diabetes was more obvious. CGM was used to evaluate the glucose change between dialysis day and non-dialysis day. The average sensor glucose concentration was 9.4mmol/L on dialysis day and 9.5mmol/L on non-dialysis day, but the average sensor glucose at night was significantly different between dialysis day and non-dialysis day. They were 8.8mmol/L and 8.4mmol/L, respectively 7, 8, 32. The utilization of CGM also helps with blood glucose control in patients. After three months of follow-up using CGM in diabetic patients on HD, their HbA1c and MG decreased, while the number of severe hypoglycemic episodes did not increase 49. As found in a study of diabetes patients with HD who received two 7-day CGM sessions: the patients were divided into baseline study period (T0) and follow-up study period (T1). After adjusting the treatment regimen according to the results of T0 period, there was no significant difference in the average glucose level, SD and %CV between the two groups on HD day and non-HD day in T1 period. Therefore, it was speculated that the application of CGM could help strengthen the blood glucose management and blood glucose control of patients 7, 34. With the use of CGM guidance to adjust the insulin regimen, the HbA1c of patients with diabetes and HD decreased from 8.4±1.0% to 7.6±1.0%, and the average glucose value decreased from 9.9±1.9 mmol/L to 8.9±2.1 mmol/L. TAR (>10 mmol/L) decreased from 41.3±21.9% to 30.1±22.4%, and TBR (<3.3 mmol/L) did not increase significantly 42. CGM monitoring allows treatment to be adjusted more frequently and individually according to blood glucose levels, thus achieving better blood glucose control without increasing the risk of hypoglycemia. CGM may be a useful tool for personalized treatment management in diabetes patients with HD 27, 42.

### Evaluating the accuracy of traditional blood glucose monitoring methods

Compared with traditional blood glucose monitoring methods, CGM can reflect the blood glucose fluctuation and changing trend of patients with diabetes and HD more comprehensively and timely, and CGM can also be used to evaluate the accuracy of its results. The study of using CGM in a patient with diabetes and HD by Yoko Narasaki et al. found that after the transition from SMBG to CGM, the patient's blood glucose control was obviously improved, and the incidence of hypoglycemia was also reduced, which may be due to the fact that using CGM to monitor blood glucose reduced the patient's burden, increased the compliance of blood glucose monitoring, and adjusted the hypoglycemic scheme more frequently 26. In patients with diabetes and HD whose average baseline blood glucose level was 8.3±2.5 mmol/L measured by CGM, they were followed up by SMBG and CGM to guide their diet or insulin therapy. At the end of the experiment, the average blood glucose level of SMBG group was 8.2±1.6 mmol/L, while that of CGM group was 7.7±1.6 mmol/L. There was no significant difference between CGM group and SMBG group in hypoglycemia time 35. CGM can also be used to evaluate the accuracy of HbA1c and GA results. The accuracy of HbA1c results is influenced by many factors in HD patients. Some studies have found that GA is more accurate in patients with diabetes and HD than glycosylated serum protein (GSP) and HbA1c, and has a stronger correlation with the 7-day average interstitial blood glucose 20, 36.

### Evaluating the efficacy and safety of anti-hyperglycemic drugs

Studies have used CGM system to evaluate the efficacy and safety of anti-hyperglycemic drugs in patients with diabetes and HD. Because most oral anti-hyperglycemic drugs are contraindications for patients with diabetes and HD, insulin therapy is the main treatment option for these patients, but insulin therapy is a strong risk factor for hypoglycemia and hyperglycemia crisis 20. At present, there is no consensus on the best blood glucose management on HD day due to conventional applications. It is suggested to avoid or reduce insulin dosage on HD day to minimize the risk of hypoglycemia. After diabetic patients on HD adjusted insulin therapy under the guidance of CGM for three months, their HbA1c decreased from 8.4% at baseline to 7.6%. At the same time, the occurrence of hypoglycemia was also reduced. The application of CGM is helpful to realize the change of insulin regimen in time according to the change of blood glucose, so as to achieve fewer symptomatic hypoglycemia attacks, reduce the average blood glucose level of patients and improve the blood glucose level as a whole 27, 36. CGM can also be used to evaluate the efficacy of dipeptidyl peptidase-4(DPP-4) inhibitor. A Japanese study found that the dosage of insulin used by patients with diabetes and HD was not only reduced, but also the incidence of asymptomatic hypoglycemia on HD day was reduced from 38.1% to 19.0%. Therefore, it is inferred that DPP-4 inhibitor may be helpful to reduce the incidence of nocturnal hypoglycemia and improve GV, Ishikawa-Tanaka and others' experiments 10, 39, 40. In addition, the application of GLP-1 agonists can also reduce the daily insulin dosage of patients with diabetes and HD, and reduce the occurrence of hypoglycemia. Among them, long-acting and short-acting GLP-1 agonists stimulate insulin secretion and reduce glucagon in a glucose-dependent manner. CGM experiments show that GLP-1 agonists can not only reduce fasting blood glucose, but also reduce postprandial blood glucose, control the decline of blood glucose during dialysis and the peak blood glucose after dialysis, and lower the GV of patients 43. Otsuka E et al. evaluated the blood glucose levels of 14 diabetic HD patients before and after switching from dulaglutide to tirzepatide through CGM. The results showed that after switching from dulaglutide to tirzepatide, the patients' TIR increased, TAR decreased, MG decreasreduced, and there was no significant difference in TBR. Therefore, they concluded that tirzepatide can significantly improve the patients' blood glucose and will not increase the risk of hypoglycemia 48.

## Limitations and challenges of CGM

However, there are some limitations in current CGM research. First, there are differences in the accuracy of equipment from different manufacturers. Second, the price of CGM is relatively expensive, which leads to limited application in some areas and may then result in regional differences in experimental results. More importantly, most studies have a small sample size. As seen from Table, the sample number of research subjects in most experimental studies is small, and some even have only a few cases. At the same time, most experiments are observational studies and may lack a control group or have a short follow-up time. All these factors limit the evaluation of the efficacy and safety of CGM 49, 56.

CGM also has some limitations. Such as sensor delays, the CGM detection value is usually delayed by 15 - 20 minutes compared to the fingertip blood glucose value. This may pose a certain risk for patients who need to adjust the treatment plan in time. For example, during hypoglycemia attacks, it may be impossible to detect and take measures immediately. A patient had hypoglycemia symptoms during dialysis, due to the delay in CGM detection, the insulin dose could not be adjusted in time, which may lead to aggravated symptoms. Another limitation is accuracy issues. The accuracy of CGM may be affected by various factors, like sensor stability and ambient temperature. For hemodialysis patients, due to the complexity of their physiological states, accuracy issues are more likely to occur. For example, in a study, the CGM monitoring results of some hemodialysis patients during dialysis were significantly different from the laboratory blood glucose results, affecting treatment decisions. The third limitation is cost barrier. CGM is relatively expensive, which is an important consideration for many patients. In some regions, medical insurance may not cover the cost of CGM, which restricts its wide application in clinical practice. Due to economic burden, many patients cannot afford CGM, affecting the effect of blood glucose management 51-55.

## Summary

Blood sugar control is a key part of self-management for diabetic patients receiving hemodialysis. CGM has many advantages in this regard. For example, real-time monitoring can reduce the incidence of hypoglycemia, lower blood sugar variability, and reduce the occurrence of cardiovascular and cerebrovascular complications.

Due to different healthcare systems and clinical practices, different countries have different degrees of recommendation for CGM. In developed countries, the healthcare system is relatively complete and the technology is advanced. The application of CGM in diabetic patients on hemodialysis is usually more active. For example, countries like the United States, on the basis of following the guidelines of the ADA, may invest more resources in promoting and applying CGM technology, including providing more comprehensive medical insurance coverage for patients and encouraging medical research institutions to conduct in-depth research to continuously improve the performance and accuracy of CGM devices. In developing countries, due to cost and technical limitations, the application of CGM is relatively limited. These regions face many challenges, such as relatively scarce medical resources and low economic development levels. However, this does not mean that developing countries cannot improve the application level of CGM. It is recommended that the governments and medical institutions in some regions take measures to improve the accessibility of CGM, such as expanding medical insurance coverage and incorporating it into routine care, so as to improve the quality of life and happiness of patients and strengthen blood glucose control to improve the prognosis of patients.

## Figures and Tables

**Table 1:** CGM application in patients with diabetes HD

Application of CGM	Researcher (time)	Sample	Population Characteristics	Wear time	CGM parameter	Accuracy (MARD), %	Study Objective	Study results
To explore the blood glucose profile	Sua Lee Etc. (2023) 7	18	18 years and older with HD time > 3 months of T1DM or T2DM	7d	MG, TIR, SD, %CV	11.0	To explore the effectiveness of CGM in blood glucose control and GV stabilization in patients with diabetes and HD	MG, HbA1c, TIR, SD and %CV were improved in HD patients with glycosuria during CGM treatment
	Tobias Bomholt Etc. (2023) 8	27	Maintenance HD T2DM	7d	Mean sensor glucose level, TIR, night mean sensor level, GV	11.0	CGM was used to examine glucose changes in T2DM patients who maintained HD	In T2DM patients undergoing maintenance HD, there is no need to distinguish between HD and non-HD day diabetes treatment regimens
	Nikita Shah Etc. (2022) 12	29	HD patients with ESDN and ESRD	6d	MG, TIR, TAR, TBR, CV, MAGE	11.0	MG and GV parameters in HD and post-HD were compared in ESDN and ESRD patients	Setting the glucose value to <180mg/dl before HD prevents greater blood glucose fluctuations during and after HD
	Yoko Narasaki (2021) 26	1	Patients with diabetes and ESRD receiving HD	20y	TIR, TAR, TBR, GV	9.0	CGM improves metabolic outcomes and quality of life in patients with ESRD diabetes	After using CGM, blood glucose monitoring compliance was improved, blood glucose parameters were improved, and hypoglycemia was reduced
	Michael Joubert (2015) 27	15	Patients with diabetes who maintain HD	5d	MG	11.0	The effect of CGM application on blood glucose control in patients with diabetes HD	CGM monitoring is associated with more frequent treatment changes those enhance blood glucose control and do not increase the risk of hypoglycemia
	F Chantrel Etc. (2014) 28	33	Patients with diabetes who maintain HD	48h	Hypoglycemia, MG, CV		CGM was used to investigate the effect of HD on blood glucose profile in patients with diabetes	During HD, MG and CV are lower and the frequency of hypoglycemia is higher
	Massimo Gai Etc. (2014) 29	12	Patients with diabetes who maintain HD	6d	High/low blood glucose	11.0	CGM was used to monitor the changes of blood glucose in patients with HD-induced diabetes	At the beginning of HD, blood glucose decreased at 3h, and hyperglycemia appeared at 2.5h after dialysis
	Sara Kazempour-Ardebili Etc. (2009) 30	17	Patients with diabetes who maintain HD	48h	Hypoglycemia, MG		CGM evaluated patients' blood glucose profiles and the differences between HD and non-HD day blood glucose profiles	Glucose values were significantly lower on HD days than in non-HD days, and the risk of asymptomatic hypoglycemia within 24 hours after HD was highest
Monitor the incidence of hypoglycemia and blood glucose fluctuations	Yasuyuki Ushiogi Etc. (2023) 31	366	CKD G3-G5 stage (including HD) and T2DM without CKD	7d	TBR	9.4	CGM assesses the prevalence and risk factors for hypoglycemia in CKD patients with or without diabetes	Decreased renal function is closely related to hypoglycemic burden in CKD patients
	Yue-ping Jin (2015) 32	92	T2DM patients, ESDN patients, ESRD patients maintained HD	72h	MG, SD, MAGE		CGM monitors blood glucose fluctuations during HD in ESDN patients	HD leads to a decrease in MG, SD, and MAGE, causing greater blood glucose fluctuations on HD days, and inaccurate HbA1c in ESDN patients.
	Hye Seung Jung Etc. (2010) 33	9	Maintain HD T2DM	144h	MAGE, SD, MG, Hypoglycemia		CGM assesses HD-associated glycemic profiles and hypoglycemia in patients with diabetes	GV is not affected by HD, which may increase the risk of hypoglycemia
	Marco Mirani Etc. (2010) 34	12	Maintain HD T2DM	2d	SD, GV, MG		Relationship between glycemic control and cardiovascular complications in patients with HD	CGM reflects an increase in GV during HD, which may be an auxiliary risk factor for cardiovascular complications
To evaluat the accuracy of traditional blood glucose monitoring methods	Maria Divani Etc. (2021) 20	37	Patients with diabetes who maintain HD	7d	MG, TIR, TAR, TBR, CV	11.0	The accuracy of GA and HbA1c in assessing blood glucose control was investigated	The accuracy of GA was higher than that of HbA1c, and neither GA nor HbA1c could accurately reflect acute shift and diurnal-day glucose variability of hypoglycemia
	Tobias Bomholt Etc. (2022) 35	66	Maintenance of HD T2DM and T2DM (eGFR>60 mL/min/1.73 m^2^)	7d	Average sensor glucose level	11.0	HbA1c and fructosamine levels were compared with CGM measurements of interstitial glucose to assess their accuracy	HbA1c underestimates plasma glucose levels in patients with HD, and CGM or fructosamine can be used as a supplementary indicator of HbA1c to reflect blood glucose levels
	Maria Divani Etc. (2018) 36	37	Patients with diabetes who maintain HD	7d	MG	11.0	CGM evaluated GA and GSP as proxies for HbA1c to reflect blood glucose control	GA was a stronger indicator of poor glycemic control for 7-day CGM than GSP and HbA1c
	Jean-Pierre Riveline Etc. (2009) 37	58	HD T2DM and non-HD T2DM were maintained	4d	MG		CGM assesses the accuracy of HbA1c and fructosamine in patients with HD diabetes	HbA1c and fructosamine are good markers of glycemic control in non-HD diabetes patients, but their accuracy is poor in HD patients
To evaluat the efficacy and safety of hypoglycemic drugs	Tobias Bomholt Etc. (2021) 38	24	T2DM patients maintained HD	7d	Hypoglycemia, GV	11.0	To evaluate the effect of liraglutide therapy on maintaining glucose variability and hypoglycemia risk in T2DM patients with HD	Liraglutide treatment increases the risk of hypoglycemic events
	Tomomi Ishikawa-Tanaka Etc. (2020) 10	31	T2DM patients maintained HD	48-168h	Hypoglycemia, GV		CGM evaluated the GV in patients during HD and the curative effect of DPP-4 inhibitors	DPP-4 inhibitors inhibit GV during HD and subsequent changes in blood glucose at night
	Marion Munch Etc. (2020) 39	65	Patients with diabetes and HD receiving insulin therapy	48 ± 6 h	MG, hypoglycemia	11.0	CGM assesses the effect of insulin in combination with vigagliptin on glycemic control in T2DM patients undergoing HD	Insulin combined with vigagliptin can improve blood glucose control and reduce insulin dosage
	Takahiro Yajima Etc. (2016) 40	21	T2DM patients maintained HD	7d	Hypoglycemia, MG, SD	11.0	The CGM evaluated the efficacy and safety of adding tiglitin to insulin therapy	Tilliptin may help reduce the total dose of HD insulin in T2DM patients and prevent hypoglycemic events

Note: CGM is continuous glucose monitoring; Mean absolute relative difference (MARD), is a measure of the difference between the predicted value and the actual value; HD is hemodialysis; T1DM is type 1 diabetes mellitus; T2DM is type 2 diabetes mellitus; MG is the average blood glucose value; Time in range (TIR) is the time when glucose is in the target range; SD is standard deviation; CV is the coefficient of variation; HbA1c is glycosylated hemoglobin; GV is blood glucose variability; Time above range (TAR) is the time when glucose is above the target range; Time below range (TBR) is the time when glucose is below the target range; Mean amplitude of glycemic excursions (MAGE) is the average blood glucose fluctuation range; GA is glycated albumin; GSP is glycosylated serum protein.
